# Cultural differences in postnatal quality of life among German-speaking women – a prospective survey in two countries

**DOI:** 10.1186/1471-2393-14-277

**Published:** 2014-08-15

**Authors:** Susanne Grylka-Baeschlin, Edwin van Teijlingen, Mechthild M Gross

**Affiliations:** Midwifery Research and Education Unit, Hannover Medical School, Carl-Neuberg-Str. 1, D-30625 Hannover, Germany; Centre for Midwifery, Maternal & Perinatal Health, Bournemouth University, Bournemouth House, 19, Christchurch Road, Bournemouth, BU1 3LH United Kingdom

**Keywords:** Mother-Generated Index, Postpartum, Wellbeing, Midwifery, Germany, Switzerland

## Abstract

**Background:**

Assessment of quality of life after childbirth is an important health-outcome measurement for new mothers and is of special interest in midwifery. The Mother-Generated Index (MGI) is a validated instrument to assess postnatal quality of life. The tool has not been applied for making a cross-cultural comparison before. This study investigated (a) responses to the MGI in German-speaking women in Germany and Switzerland; and (b) associations between MGI scores on the one hand and maternity and midwifery care on the other.

**Methods:**

A two-stage survey was conducted in two rural hospitals 10 km apart, on opposite sides of the German-Swiss border. The questionnaires included the MGI and questions on socio-demographics, physical and mental health and maternity care, and were distributed during the first days after birth and six weeks postpartum. Parametric and non-parametric tests were computed with the statistical programme SPSS.

**Results:**

A total of 129 questionnaires were returned an average of three days after birth and 83 in the follow-up after seven weeks. There were no statistically significant differences in the MGI scores between the German and the Swiss women (p = 0.22). Significantly more favourable MGI scores were found associated with more adequate information during pregnancy (p = 0.02), a more satisfactory birth experience (p < 0.01), epidural anaesthesia (p < 0.01), more information (p = 0.01) and better support (p = 0.02) during the time in hospital and less disturbed sleep (p < 0.01). Significantly lower MGI scores were associated with the presence of a private doctor during birth (p = 0.01) and with exclusive breastfeeding during the first postnatal days (p = 0.04).

**Conclusion:**

The MGI scores of these German-speaking women were higher than those in other studies reported previously. Thus the tool may be able to detect differences in postnatal quality of life among women with substantially divergent cultural backgrounds. Shortcomings in maternity and midwifery care were detected, as for example the inadequate provision of information during pregnancy, a lack of individualised postpartum care during the hospital stay and insufficient support for exclusively breastfeeding mothers. The MGI is an appropriate instrument for maternity care outcome measurement in cross-cultural comparison research.

## Background

Assessing postnatal quality of life for cross-cultural comparison provides information on gaps in maternity care. Quality of life can be considered both as a health care outcome and as a maternity care outcome that widens the scope of the traditional indicators focusing on morbidity, mortality and life expectancy [1]. However, there is no uniform definition of the concept of quality of life [2]. Quality of life is subjective by nature and is associated both with health-related and with non-medically related factors [3]. Women experience changes in their quality of life during pregnancy and the postpartum period [4–6].

Due to the lack of uniform definition and the subjective nature of quality of life, its assessment is challenging. Widely used tools to assess postnatal quality of life include the Short Form Health Survey SF-36 [7] and the World Health Organization’s Quality of Life tool WHOQOL-100 and the WHOQOL-BREF [8]. However, neither instrument is specifically designed for the postnatal period. The Mother-Generated Index (MGI) was developed by Symon et al. [9] as the first tool designed to assess postnatal quality of life. The tool does not provide a predefined checklist of problems, and thus respects the subjective nature of quality of life and measures aspects of it as the mother perceives them [9]. The original English version of the MGI was validated with 102 Scottish women [10]. The MGI has been translated into different languages [11–13] however not previously into German, and has never before been used for cross-cultural comparison. Associations between variables relating to pregnancy, labour, birth, the postpartum period and care processes on the one hand and differences in postnatal quality of life on the other have not been investigated before.

The relationship between maternity and midwifery care and postnatal quality of life has remained unclear. Hildingsson et al. [14] found dissatisfaction with antenatal care in respect of the information provided before the birth on the situation to be expected after it. Gürber et al. [15] investigated the relation between mothers’ reports of caregiver support, their subjective birth experiences and the changes in acute stress symptoms and postpartum depression symptoms one to three weeks postpartum. Women who reported more favourably on caregiver support had better subjective birth experiences, and these were associated with less acute stress and postpartum depression symptoms, which have been found to be linked to postpartum quality of life [16]. Benoit et al*.*[17] also noticed that satisfaction with the birth experience was linked to a lower incidence of depression after birth. Reports on the impact of mode of delivery on postnatal quality of life are contradictory. Whereas Symon et al. [9] found no association, other authors using different tools found a lower quality of life after a caesarean section [18, 19]. A systematic review of the literature failed to demonstrate that universal postpartum support is effective in improving parenting, maternal mental health, maternal quality of life or maternal physical health [20]. In one case, a feeling that postpartum care was inadequate was reported to have arisen out of the fact that nurses and new mothers had differing perceptions of maternal needs [21]. In other cases, women have been found to be very critical about postnatal care and have reported insensitivity, inconsistent advice, not very helpful support and advice on feeding the baby, inadequate assessments and care, lack of emotional support, too busy hospital staff and too few home visits [22–25]. Better communication between mothers and health care professionals was found to be directly associated with a higher level of satisfaction with postnatal care [26]. Shaw et al. [20] suggested that low-income primiparous women and women at high risk of family dysfunction may profit especially from postnatal care.

Germany and Switzerland are two neighbouring countries in central Europe with German as at least one of their national languages. Both countries have well developed health care systems [27, 28], and in both countries all births are attended by a midwife [29, 30]. A major difference is the length of midwifery home support. Postpartum home visits are usually covered by the health insurance during the first eight weeks after birth in Germany, but only during a period of ten days in Switzerland [31, 32]. Furthermore, German parents benefit from 14 months of paid parental leave (in total for both parents) whereas Swiss women are entitled to only 14 weeks of maternity leave [33–35].

The aims of this study were (a) to investigate how the primary and secondary MGI scores as well as a number of identified areas of life differed between women giving birth in the German and the Swiss hospital; and (b) to correlate MGI scores with variables relating to antenatal, intrapartum and postpartum care.

## Method

The study comprised a prospective cross-cultural two-stage survey, carried out in two rural hospitals located in the south of Germany and the north of Switzerland. The two hospitals were situated in the same geographical area but on opposite sides of the border, and had similar numbers of births per year.

### Study instruments

**Figure 1:**
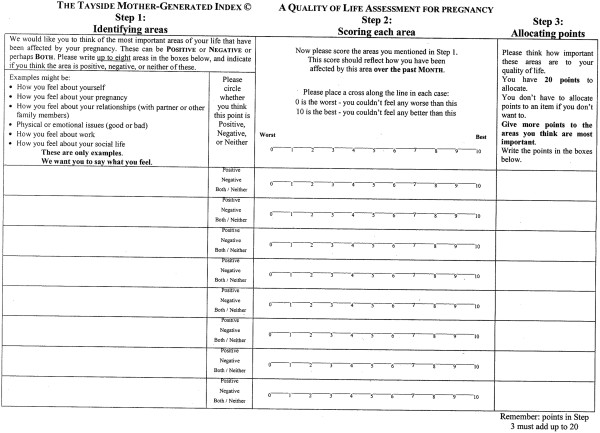
**The original English MGI form developed by Andrew Symon.**

The Mother-Generated Index (MGI) is a single sheet questionnaire involving three steps (Figure 1). A primary and a secondary score can be calculated, each ranging from zero to ten, with ten indicating the highest possible quality of life [9]. The primary score is computed with the mean of the values on the visual analogue scales in step two of the MGI Index, whereby women scored the areas of life identified in step one. The secondary score is calculated with the sum of the same values on the visual analogue scales in step two multiplied by the allocated spending points of step three and divided by 20. Secondary scores therefore take into consideration the importance of the identified and scored areas of life for the woman.

The MGI is an instrument designed to be administered alongside other measures [36]. Two questionnaires were therefore developed [37] to be administered during the first days after birth and six weeks postpartum. Large maternity surveys published in the literature were examined in detail in search of questions relevant to the purpose of the study [38, 39]. Both questionnaires included the MGI, the Hospital Anxiety and Depression Scale (HADS), the Postnatal Morbidity Index (PMI), and questions on labour and birth experience [40, 38, 41, 42]. In addition, the first questionnaire addressed perinatal aspects and maternity care during pregnancy, labour, birth and the time in hospital. The second questionnaire included additional questions concerning postpartum support at home. The surveys were forward and back translated using a multistep method to ensure the quality of the translation [43], and then pilot-tested with ten women during the postpartum period [11].

### Study process

All women giving birth in the selected German and Swiss hospitals between 1 October and 15 December 2012 were invited to participate in the study, unless they had insufficient knowledge of the German language or their babies were referred to a neonatal care unit. A total of 226 women were eligible to participate, 131 in Germany and 95 in Switzerland. In total 129 women participated (response rate 57.1%), 77 in the German subsample and 52 in the Swiss subsample. The first questionnaire was completed on average three days after birth (range 1–28 days). Of these 129 women, 98 (76%) agreed to have a second questionnaire sent to them by post at the end of the postpartum period [44], which is generally after about six weeks. The follow-up survey was completed after an average of seven weeks (range 5.5-15 weeks). The rate of response to the second questionnaire was 84.7%, representing 83 participants, 45 in the German subsample and 38 in the Swiss subsample.

### Ethical considerations

The Ethics committee of Hannover Medical School (Germany) approved the research proposal. Approval was also obtained from the relevant Swiss Canton ethics committee. The women concerned consented to participate after being given verbal and written information on the aim of the study and the study process, including the fact that their participation was voluntary and that they had the right to withdraw at any time without suffering any disadvantage. The collected data were treated as strictly confidential.

### Data analysis

Descriptive analysis was carried out on socio-demographic and perinatal data and on the MGI scores. Comparisons of socio-demographic and perinatal data were computed with Student’s t-test, the chi-squared test or the Fisher’s exact test. Student’s t-test was used to compare the normally distributed MGI primary scores, and the Mann–Whitney U-test for the comparison of the secondary scores with their skewed distribution. Associations between the MGI scores and questions relating to socio-demographic and perinatal care factors were computed using Pearson’s correlation coefficient r, Student’s t-test and the Mann–Whitney U-test. Multiple linear regression models were computed with the MGI primary scores as dependent variables. Results were considered to be statistically significant where p < 0.05. Statistical analyses were made using the statistical programme SPSS version 20.

## Results

### Characteristics of the participants

The average age of the participants was 30.3 years (Table 1). Women who gave birth at the German hospital (n = 77) were significantly younger than women who gave birth at the Swiss hospital (n = 51; 29.3 years versus 31.8 years, p < 0.01). The percentage of primiparous women was 62.3% in the German hospital compared to 46.2% in the Swiss hospital (p = 0.07). Excluding planned caesarean sections, duration of labour was self-reported to be significantly longer by women who gave birth in the German hospital compared to the Swiss one (11.00 hours versus 6.56 hours, p < 0.01). Self-estimated labour durations for vaginal births, excluding all caesarean sections, were also significantly longer in the German subsample (10.60 hours hours versus 6.19 hours, p = 0.02). Caesarean section rates were significantly higher in the Swiss unit than in the German unit (44.2% versus 25.0%, p = 0.02) whereas epidural anaesthesia was more often used in the German hospital (50.8% versus 20.0%, p < 0.01).

**Table 1:** **Characteristics of the participants at the German and Swiss hospitals**

Variable		Total sample	German hospital	Swiss hospital	Test	p-value
**Age**	n	128	77	51		
Mean	Years	30.30	29.32	31.78	t-test = −2.94	p < 0.01*
Range		16 - 43	16-43	22-40		
SD		4.78	5.13	3.78		
**Primiparous**	n	129	77	52		
	% (n)	55.8 (72)	62.3 (48)	46.2 (24)	χ^2^ = 3.30 df = 1	p = 0.07
**Length of labour including unplanned c-section**	n	100	64	36		
Mean	Hours	9.40	11.00	6.56	t-test = 2.72	p < 0.01*
Range		0.25 - 72.00	0.25 - 72.00	0.25 - 28.00		
SD		9.43	10.73	5.57		
**Length of labour only vaginal births**	n	86	57	29		
Mean	Hours	9.11	10.60	6.19	t-test = 2.44	p = 0.02*
Range		0.25 - 72.00	0.25 - 72.00	0.25 - 28.00		
SD		9.86	11.19	5.58		
**Mode of birth**	n	128	76	52		
Vaginal birth	% (n)	67.2 (86)	75.0 (57)	55.8 (29)	χ^2^ = 5.18	p = 0.02*
Caesarean section	% (n)	32.8 (42)	25.0 (19)	44.2 (23)	df = 1	

*Significant differences at p <0.05.

### Comparison of MGI scores between German and Swiss women

Three days after birth, the average primary score of the Mother-Generated Index (MGI) was 7.20 and was more favourable for women who gave birth in the German hospital than for those at the Swiss hospital, though the difference was not significant (7.34 versus 7.00, p = 0.22) (Table 2). The secondary scores averaged 7.84, so were higher than the primary scores. Again, there was no significant difference between the subsamples (German unit: 7.99, Swiss unit: 7.62, p = 0.07). Women identified an average of 5.10 areas of life affected by having a baby.

**Table 2:** **MGI scores three days postpartum by country**

MGI scores three days after birth		Total sample	German hospital	Swiss hospital	Test	p-value
**MGI primary scores**	n	121	72	49		
	Mean	7.20	7.34	7.00	t-test = 1.24	p = 0.22
	Range	3.20 - 10.00	3.20 - 10.00	4.00 - 10.00		
	SD	1.50	1.50	1.51		
**MGI secondary scores**	n	114	69	45		
	Mean	7.84	7.99	7.62	U = 1239.00	p = 0.07
	Range	3.20 - 10.00	3.20 - 10.00	5.27 - 9.80		
	SD	1.43	1.52	1.27		
**Number of areas of life**	n	125	75	50		
	Mean	5.10	4.88	5.42	t-test = −1.74	p = 0.08
	Range	1 - 8	1 - 8	1 - 8		
	SD	1.71	1.59	1.84		

Seven weeks after birth, the picture was similar (Table 3). Primary and secondary scores were slightly, but not significantly, more favourable for women who gave birth at the German hospital than for those who gave birth at the Swiss hospital (primary scores: 6.92 versus 6.66, p = 0.43; secondary scores: 7.47 versus 6.95, p = 0.17). Women in both units identified similar numbers of areas of life. MGI primary and secondary scores were lower seven weeks postpartum compared to the values assessed directly after birth (primary score 6.80 versus 7.20; secondary score 7.23 versus 7.84).

**Table 3:** **MGI scores seven weeks postpartum by country**

MGI scores after 7 weeks		Total sample	German hospital	Swiss hospital	Test	p-value
**MGI primary scores**	n	82	44	38		
	Mean	6.80	6.92	6.66	t-test = 0.80	p = 0.43
	Range	3.33 - 9.50	3.67 - 9.50	3.33 - 9.50		
	SD	1.48	1.34	1.63		
**MGI secondary scores**	n	72	39	33		
	Mean	7.23	7.47	6.95	U = 521.00	p = 0.17
	Range	2.86 - 9.75	3.45 - 9.65	2.86 - 9.75		
	SD	1.76	1.62	1.90		
**Number of areas of life**	n	82	44	38		
	Mean	5.70	5.55	5.87	t-test = −1.02	p = 0.31
	Range	3 - 8	3 - 8	3 - 8		
	SD	1.44	1.39	1.49		

### Comparison of maternity care between German and Swiss subsamples

Women estimated the quality of information they received during pregnancy on five-point Likert scales for the sub-questions regarding information about pregnancy, birth, breastfeeding and life with a baby in a very similar way (Table 4). All births in Germany and Switzerland are attended by a midwife. For unknown reasons, one woman in each country indicated that no midwife attended her birth. Most of the births in both countries are also attended by a medical doctor, either a doctor associated with the hospital or a private doctor. A feature occurring significantly more often in the Swiss than in the German hospital was that private doctors attended births (37.3% versus 6.8%, p < 0.001). Seven weeks postpartum, women who gave birth at the German hospital assessed their birth experiences significantly more highly on visual analogue scales ranging from zero to ten than did women who gave birth at the Swiss hospital (7.89 versus 6.71, p = 0.04). Information provided by the hospital staff during the time in hospital, assessed with the sum of the values on five-point Likert scales for the sub-questions “hospital stay”, “baby care”, “breastfeeding” and “life with a baby” was evaluated significantly more favourably in the Swiss than in the German hospital (13.92 points versus 12.52 points, p < 0.001). Support from the hospital, also assessed with the sum of values on five-point Likert scales for the sub-questions “personal hygiene”, “baby care” and “breastfeeding”, was evaluated similarly (German hospital 11.82, Swiss hospital 12.93, p = 0.03). Significantly more women exclusively breastfed their children in the Swiss than in the German hospital (69.2% versus 45.5%, p < 0.01).

**Table 4:** **Maternity care related variables by country, assessed after three days or seven weeks**

Maternity care related variable		Total sample	German hospital	Swiss hospital	Test	p-value
**Information during pregnancy, 3 days**	Mean	15.91	15.88	15.94	U = 1712.50	p = 0.49
	Range	8 - 20	8 - 20	8 - 20		
**Information during pregnancy, 7 weeks**	Mean	15.57	15.58	15.55	U = 821.50	p = 0.92
	Range	8 - 20	9 - 20	8 - 20		
**Epidural, 3 days**	n	105	65	40		
Yes	% (n)	39.0 (41)	50.8 (33)	20.0 (8)	χ^2^ = 9.85	p < 0.01*
**Midwife attended birth, 3 days**						
Yes	% (n)	98.4 (123)	98.6 (73)	98.0 (50)	Fischer’s exact test	p = 1.00
**Hospital doctor attended birth, 3 days**						
Yes	% (n)	76.0 (95)	93.2 (69)	51.0 (26)	χ^2^ = 29.57	p < 0.001*
**Private doctor attended birth, 3 days**						
Yes	% (n)	19.2 (24)	6.8 (5)	37.3 (19)	χ^2^ = 18.10	p < 0.001*
**Labour experience, 3 days**	Mean	3.26	3.22	3.31	t-test = −0.19	p = 0.85
	Range	0 - 9.4	0 - 8.4	0 - 9.4		
**Labour experience, 7 weeks**	Mean	4.37	4.64	4.07	t-test = 0.98	p = 0.33
	Range	0 - 9.5	0-8.0	0 - 9.5		
**Birth experience 3 days**	Mean	6.83	7.02	6.55	t-test = 0.99	p = 0.32
	Range	0 - 10.0	1.4 - 10.0	0 - 10.0		
**Birth experience 7 weeks**	Mean	7.36	7.89	6.71	t-test = 2.08	p = 0.04*
	Range	0 - 10.0	2 - 10	0 - 10		
**Information from hospital staff, 3 days**	Mean	13.09	12.52	13.92	U = 1141.00	p < 0.001*
	Range	6 - 15	6 - 15	9 - 15		
**Information from hospital staff, 7 weeks**	Mean	11.41	10.72	12.20	U = 536.00	p = 0.01*
	Range	5 - 15	5 - 15	8 - 15		
**Support from hospital staff, 3 days**	Mean	12.26	11.82	12.93	U = 1380.50	p = 0.03*
	Range	5 - 15	5 - 15	6 - 15		
**Exclusively breastfeeding, 3 days**		129	77	52		
Yes	% (n)	55.0 (71)	45.5 (35)	69.2 (36)	χ^2^ = 7.09	p < 0.01*

*Significant differences at p <0.05.

### Associations between MGI scores and perinatal and midwifery care

MGI scores were not significantly associated either with age, parity or mode of birth. Higher primary scores correlated with more information during pregnancy in the Swiss subsample (p < 0.001), and as a result in the whole study population (p = 0.02); in the German subsample, by contrast, no correlation was found (p = 0.72) (Table 5). Better birth experiences were associated with higher scores after seven weeks, but not three days postpartum. These associations were significant for the whole study population (primary score: p < 0.01; secondary score: p < 0.01) and the German subsample (primary score: p < 0.01; secondary score: p < 0.01), but not for the Swiss subsample. Directly after birth, epidural anaesthesia was positively and significantly associated with higher MGI secondary scores (p = 0.03). Seven weeks postpartum, primary and secondary scores were positively and significantly associated with epidural anaesthesia in the whole study population and in the Swiss subsample (primary score total sample: p < 0.01).

**Table 5:** **Association between MGI scores and variables relating to care during pregnancy and labour**

Associations between MGI scores and care during pregnancy & labour		Total sample	German hospital	Swiss hospital
**Information during pregnancy** ^**1**^				
MGI primary score **3 days**	n	117	68	49
	r, *p*	**0.21,** 0.02*	−0.05, *0.72*	**0.49,** <0.001*
MGI secondary score **3 days**	n	110	65	45
	r, *p*	0.16, *0.10*	−0.02, *0.89*	**0.43,** <0.01*
MGI primary score **7 weeks**	n	81	44	37
	r, *p*	0.12, *0.29*	−0.04, *0.82*	0.24, *0.15*
MGI secondary score **7 weeks**	n	72	39	33
	r, *p*	0.10, *0.43*	0.03, *0.87*	0.16, *0.38*
**Birth experience** ^**1**^				
MGI primary score **3 days**	n	118	71	47
	r, *p*	0.12, *0.20*	0.04, *0.72*	0.20, *0.18*
MGI secondary score **3 days**	n	112	68	44
	r, *p*	0.17, *0.08*	0.22, *0.08*	0.06, *0.69*
MGI primary score **7 weeks**	n	81	44	37
	r, *p*	**0.33,** ***<0.01***	**0.41, <** ***0.01***	0.26, *0.12*
MGI secondary score **7 weeks**	n	71	39	32
	r, *p*	**0.34,** ***<0.01***	**0.44, <** ***0.01***	0.23, *0.20*
**Epidural** ^**2**^				
MGI primary score **3 days**	n	98	61	37
	t-test, *p*	−1.90, *0.06*	−1.01, *0.32*	−1.53, *0.14*
MGI secondary score **3 days**	n	94	58	36
	U, *p*	**770.00,** 0.03*	340.50, *0.21*	64.00, *0.13*
MGI primary score **7 weeks**	n	72	39	33
	t-test, *p*	**−3.04,** <0.01*	−1.26, *0.22*	**−3.16,** <0.01*
MGI secondary score **7 weeks**	n	64	34	30
	U, *p*	**278.00,** 0.03*	136.50, *1.00*	**17.00,** <0.01*
**Private doctor** ^**2**^				
MGI primary score **3 days**	n	117	69	48
	t-test, *p*	1.95, *0.05*	−0.98, *0.33*	**2.61,** 0.01*
MGI secondary score **3 days**	n	111	66	45
	U, *p*	716.00, *0.14*	88.00, *0.84*	206.00, *0.45*
MGI primary score **7 weeks**	n	80	42	38
	t-test, *p*	−0.35, *0.73*	−0.93, *0.36*	−0.31, *0.76*
MGI secondary score **7 weeks**	n	70	37	33
	U, *p*	387.00, *0.94*	13.00, *0.64*	116.00, *0.61*

^1^Assessed three days and seven weeks after birth. The associations were computed with the MGI scores at the same time of assessment ^2^assessed in the questionnaire three days after birth. The associations were computed with the MGI scores at both stages of assessment, *significant associations at p <0.05.

In the German subsample, secondary scores correlated significantly, weakly and positively with the information provided by the hospital staff (p = 0.01) (Table 6). The support women received from hospital staff during their time in hospital was significantly associated with secondary scores in the whole study population (p = 0.02) and in the German subsample. After seven weeks, significant weak positive correlations were found between primary and secondary scores and the longest periods of rest or sleep (primary score total sample: p < 0.01). However, the average time spent asleep per night was not found to be associated with MGI scores (primary score total sample: p = 0.65).

**Table 6:** **Association between MGI scores and variables relating to postpartum care**

Associations between MGI scores and perinatal care		Total sample	German hospital	Swiss hospital
**Information from hospital staff** ^**1**^				
MGI primary score **3 days**	n	115	67	48
	r, *p*	0.11, *0.26*	0.14, *0.24*	0.18, *0.23*
MGI secondary score **3 days**	n	108	64	44
	r, *p*	0.18, *0.06*	**0.32,** 0.01*	0.05, *0.73*
**Support from hospital staff** ^**1**^				
MGI primary score **3 days**	n	115	67	48
	r, *p*	0.13, *0.17*	0.14, *0.25*	0.17, 0*.25*
MGI secondary score **3 days**	n	109	65	44
	r, *p*	**0.22,** 0.02*	**0.29,** 0.02*	0.17, *0.27*
**Exclusive breastfeeding** ^**1**^				
MGI primary score **3 days**	n	119	70	49
	t-test, *p*	1.48, *0.14*	0.35, *0.73*	1.47, *0.15*
MGI secondary score **3 days**	n	112	67	45
	U, *p*	1307.50, *0.15*	546.50, *0.96*	**124.00,** 0.04*
**Longest rest or sleep** ^**2**^				
MGI primary score **7 weeks**	n	80	42	38
	r, *p*	**0.31,** <0.01*	0.28, *0.08*	**0.37,** 0.02*
MGI secondary score **7 weeks**	n	70	37	33
	r, *p*	**0.28,** 0.02*	0.24, *0.15*	**0.39,** 0.03*

^1^Assessed three days after birth, ^2^assessed seven weeks after birth, *significant associations at p <0.05.

Higher MGI primary scores were negatively associated with the presence of a private doctor during birth in the Swiss subsample (p = 0.01) directly after birth but not after seven weeks (Table 5). For women giving birth at the Swiss hospital, secondary scores were negatively associated with exclusive breastfeeding (p = 0.04, Table 6).

### Multiple regression models for the MGI primary scores

In the whole study population, the only significant predictor three days after birth was if a doctor from the hospital and not a private doctor attended a birth (p < 0.01). In the German subsample, significant predictors were the support from the hospital staff (p = 0.046) and the Hospital Anxiety and Depression Scale (HADS) depression subscore (p = 0.03). In the Swiss subsample, by contrast, information during pregnancy (p = 0.01), the presence of a private doctor (p = 0.01) and exclusive breastfeeding (p = 0.04) were significant predictors for the MGI primary scores. Seven weeks after birth, epidural anaesthesia was the only predictor for MGI primary scores (p < 0.01) in the whole study population.

## Discussion

The associations between the Mother-Generated Index (MGI) scores, on the one hand, and variables relating to pregnancy, labour, birth, the postpartum period and care processes, on the other, were generally inconsistent for both scores and all samples. Nevertheless, the results provide relevant information on gaps in and improvement possibilities for perinatal and midwifery care.

### Cultural differences in MGI scores

As this was the first study using the MGI directly after birth, there were no comparative values for this timing of the investigation. MGI primary and secondary scores directly after birth and seven weeks postpartum did not significantly differ between German and Swiss women. It was however possible to compare the scores of the follow-up after seven weeks with the results of the original Scottish study with 103 participants [10], indicating an average primary score of 6.05 [45]. Primary scores of 6.92 in the German sample and of 6.60 in the Swiss sample were both higher than the scores in the Scottish study, and the differences to the Scottish studies were higher than the difference between the German and the Swiss sample. By contrast, the Indian study of Nagpal et al. [11], with 195 participants completing the MGI during the first six months following birth, recorded primary scores of 3.6 and secondary scores of 2.9; and the Iranian study of Khabiri et al. [13] with 96 participants completing the MGI during the first six weeks after birth showed a mean primary score of 5.38 and secondary score of 6.47. These scores were all lower than the values in the German and the Scottish study. It is difficult to assess whether this variation reflects differences in the perception of the concept of quality of life, in the evaluation of high or low values or in the difference regarding the timing of administration. However, it may be deduced that the MGI identifies cultural differences in postnatal quality of life in settings where these are substantially greater than between Germans and Swiss living in the same geographical area. Further research comparing MGI scores in more divergent settings is needed.

### Associations between MGI scores and perinatal care

MGI scores were not found to be associated with mode of birth, which corresponds to the results of the Scottish study by Symon et al. [9]. It is in contrast to studies using other tools to assess postnatal quality of life [18, 19]. This could be related to the inconsistencies in the definitions of quality of life [2], indicating that different quality of life tools may measure different concepts of quality of life, or the characteristic and the size of the German-speaking sample. Further studies investigating the association between MGI score and mode of birth are needed.

The receipt of information during pregnancy was appraised very similarly in both hospitals. However, women in the Swiss sample who rated the information furnished during pregnancy more highly had significantly higher MGI scores than women who were less satisfied with the information given during pregnancy, as was also confirmed in the regression model. A lack of information about pregnancy and the feeling of not being taken seriously have been found to be the main factors contributing to dissatisfaction with antenatal care [14]. The associations between satisfaction with information during pregnancy and postnatal quality of life found in the Swiss sample represent a further indication that the quality of antenatal care should be reconsidered with a view to its effects on postnatal quality of life. Antenatal care should not only consist of a physical check-up, time and willingness to answer questions and provide information are necessary as well. In the German subsample, by contrast, this significant association was not found. This might be because of differences in the expectations toward antenatal care or because of differences in gathering information, indicating a cultural difference in the requirement for information during pregnancy in order to have a feeling of wellbeing after birth.

Labour and birth experiences were not associated with postnatal quality of life directly after birth. After about seven weeks, however, birth experience was so associated. This shows that birth experience had a more significant impact on postnatal quality of life than labour experience. Improvements in intrapartum care should concentrate on personal expectations, support from caregivers, the quality of the caregiver relationship and involvement in decision-making, because these aspects were found to have a higher impact on the birth experience than pain [46]. However, the present study also found a higher postnatal quality of life for women who had undergone epidural anaesthesia, and the differences in quality of life between women who had had an epidural and those who had not became more distinct after seven weeks than during the first week postpartum. This could indicate that pain, pain relief methods or expectations about the pain that has to be borne are nevertheless important if women are to experience a feeling of wellbeing after birth. However, because the offering of epidural anaesthesia cannot be recommended unreservedly, in view of its side-effects [47], the effectiveness of alternative pain relief methods should be made the object of further research. The association between the presence of a private doctor during birth and the lower postnatal quality of life in the Swiss sub-sample found in the current study was surprising. Furthermore, the presence of a private doctor was a significant predictor in the regression model. In Switzerland, having a private doctor in attendance is very popular. Such private doctors are mostly consultants in obstetrics and gynaecology who are known to the women from antenatal care and booked to come to the hospital specially to attend them during the birth of their child. The attendance of private doctors may raise women’s expectations about their care which are probably not completely satisfied. This result must be interpreted with caution. It is worth pointing out that the private doctor system is very expensive and private maternity care was found to be associated with an increased risk of obstetric intervention [48, 49]. This needs to be considered in future discussions.

Women who evaluated information and support during their time in hospital less favourably, or who exclusively breastfed their children and required adequate breastfeeding support, showed a reduced postnatal quality of life. These associations were stronger for the MGI secondary scores, for which there is no apparent explanation, and also stronger in the German unit where information and support were evaluated less favourably, respectively in the Swiss unit where exclusive breastfeeding rates were higher. Taking into consideration that recognising the needs of new mothers has been found to be necessary to enhance postpartum care [21] and that the hospital environment influences women’s postpartum experience [50], the findings of the current study support Shaw et al. [20] conclusion that routine postpartum care to unselected low-risk women does not appear to improve any of the maternal outcomes. The World Health Organization [51] proposed that postnatal care should be individualised and that women and their babies should be placed at the focus of care provision. Maternity care during the first few days after birth should be reorganised in order to individualise support and information, which is a challenge in view of the staffing constraints that frequently prevail. In the later postpartum period, postnatal quality of life was associated with the longest periods of rest or sleep enjoyed by the women, but not with the average hours of sleep per night. Sleep quality could be improved with counselling, as well as with holistic and individualised postnatal care which includes positively supporting essential human needs such as sleep. German midwives are able to support women for a longer period of time than Swiss midwives are [31, 32]. Nevertheless, MGI scores seven weeks postpartum were not significantly higher in the German than in the Swiss subsample, and further research including assessment several months after birth would be necessary to completely understand the impact of the length of midwifery support on postnatal quality of life.

### Strengths and weaknesses

A key strength of the current study was that it was conducted in two countries, Germany and Switzerland, and that the samples should have a representative character for the hospitals selected, since all women giving birth during the determined timespan were invited to participate. However, socio-demographic and perinatal para-meters could not be compared to annual figures of the hospitals, because of missing accessibility and thus, selection bias because of the relatively short observation period cannot be excluded. A further strength was that all women were furnished with detailed oral and written information by the same investigator (a native German speaker). Thus all the women had very similar information and verbal explanations, which ensured a consistent initial situation for the whole sample. Furthermore, all three maternity periods, pregnancy, labour and birth, and also the postpartum period were investigated.

Because of the small size of the hospitals and the response rate of just below 60%, the sample was relatively small, with 129 women participating during the first week after birth and 83 after seven weeks. The differences in MGI scores which were found might have proved statistically significant if the sample had been larger. This is a common problem in smaller studies which provide some indications but frequently lack statistical significance [52]. However, the study is currently the third largest study using the MGI and no other study to date has conducted a follow-up. The follow-up allowed the evolution of postnatal quality of life and its association with perinatal care over the first seven weeks after birth to be investigated.

The self-completion of the MGI by women who were using the tool for the first time was also a limitation. Mothers in the Polish study of Nowakowska-Glab et al. [53], the only one including the MGI and using the same approach as the current study, concluded that women could not self-complete the MGI. The present study found that 70% of the MGI forms were completed absolutely correctly. Using minor adjustment, it was possible to calculate the majority of the MGI scores (between 87% and 99%). The availability of scores was therefore satisfactory, but the error rates could indicate that not all women understood exactly what to do. This could have led to inaccurate scores because of misunderstandings of the task.

## Conclusion

The translated Mother-Generated Index (MGI) did not show differences in scores as between women giving birth in a German and in a Swiss hospital. However, there is evidence that the instrument may be able to detect differences in postnatal quality of life in more heterogeneous samples. Further research using the MGI with larger samples is needed.

The associations between the MGI scores, on the one hand, and variables relating to pregnancy, labour, birth, the postpartum period and care processes, on the other, showed up aspects of maternity care that need to be improved. The present study demonstrated the potential ability of the instrument to detect possibilities for improvement in maternity and midwifery care.

## Author’s information

Susanne Grylka-Baeschlin is a midwife with an MSc in midwifery and is a PhD student. She is currently working as a scientific assistant at Hannover Medical School. Edwin van Teijlingen PhD is a medical sociologist and professor at the Centre for Midwifery, Maternal & Perinatal Health at Bournemouth University. Mechthild M. Gross, PD Dr., is a nurse and midwife and psychologist and head of the Midwifery Research and Education Unit at Hannover Medical School.
